# Effects of Aerobic Training Versus Behavioral Intervention to Increase Physical Activity for Disease Control in Patients With Asthma: Protocol for a Randomized Trial

**DOI:** 10.2196/78603

**Published:** 2025-10-30

**Authors:** David Halen Araujo Pinheiro, Ronaldo Aparecido da Silva, Adriana Claudia Lunardi, Vitoria Zacarias Cervera, Regina Maria Carvalho-Pinto, Fabiano Francisco de Lima, Celso R F Carvalho

**Affiliations:** 1 Department of Physical Therapy, School of Medicine University of São Paulo São Paulo Brazil; 2 Pulmonary Division, Heart Institute (InCor), Clinics Hospital Universidade de São Paulo São Paulo Brazil

**Keywords:** asthma, physical activity, physical exercise, sedentarism, quality of life

## Abstract

**Background:**

Aerobic training (AT) and behavioral intervention (BI) aimed at increasing physical activity provide numerous benefits to patients with asthma. However, the comparison between the two interventions in the clinical control of this disease is poorly understood.

**Objective:**

This study aims to compare the effects of AT and BI on disease control in people with moderate to severe asthma.

**Methods:**

This is a randomized 2-arm clinical trial with a blinded evaluation. The study will include 56 physically inactive adults with uncontrolled asthma despite optimized medication. Eligible patients will be randomized into either the AT group or the BI group. AT will be performed on a treadmill for 8 weeks (2 sessions per week, 45 minutes each), and the intensity will be determined by the maximum heart rate established in the cardiopulmonary exercise test. BI will be based on social cognitive theory and behavioral change stages that will last 8 weeks (1 session per week, 90 minutes per session). All interventions will last a total of 12 hours. Assessments will be conducted pre- and postintervention and again 16 weeks later and will include physical activity in daily life (PADL; assessed using triaxial accelerometry) level, body composition (measured using octopolar bioimpedance), barriers to PADL (questionnaire), clinical asthma control (Asthma Control Questionnaire), quality of life (Asthma Quality of Life Questionnaire), anxiety and depression levels (Hospital Anxiety and Depression Scale), and number of exacerbations. Time and group interactions will be evaluated using 2-way repeated measures ANOVA. The significance level will be set at *P*<.05.

**Results:**

As of October 2025, 52 participants had been recruited. This study was funded by the São Paulo Research Foundation and the National Council for Scientific and Technological Development. Clinical trial registration was granted in May 2022. Recruitment and data collection for the trial are ongoing, and the results of this study are expected to be available by the end of December 2026.

**Conclusions:**

This is the first study to compare the effects of AT versus BI on increasing physical activity for clinical asthma control. Therefore, the results obtained in the proposed protocol may provide essential information for health care professionals when recommending these approaches to people with asthma.

**Trial Registration:**

ClinicalTrials.gov NCT05364632; https://clinicaltrials.gov/study/NCT05364632

**International Registered Report Identifier (IRRID):**

DERR1-10.2196/78603

## Introduction

**Overview**

Asthma is a heterogeneous disease characterized by chronic airway inflammation [1]. It is defined by respiratory symptoms, including shortness of breath, wheezing, chest tightness, and variable expiratory airflow limitation [1]. It is a global problem, and its incidence and prevalence are increasing [2]. Asthma is the second most common chronic respiratory disease and affects approximately 300 million people worldwide [3]. In 2019, 262 million people were diagnosed with asthma, and 461,000 died from the disease [3].

Asthma control strategies include pharmacological and nonpharmacological treatments [1]. A structured and supervised aerobic exercise program, such as aerobic training (AT), is a nonpharmacological behavioral treatment known to improve asthma control [4-7]. Behavioral intervention (BI), aiming to increase physical activity in daily life (PADL), is also part of the nonpharmacological treatment [8]. Both interventions improve the patient’s quality of life and asthma symptoms and help minimize exacerbation episodes [7-9].

In 2020, the World Health Organization [10] recommended that adults perform 150 to 300 minutes of moderate-intensity aerobic physical activity weekly to achieve health benefits. People with asthma are less physically active than their healthy counterparts [11]; thus, developing strategies to modify behavior, increase physical activity, and reduce sedentary time among people with asthma is important [12]. Freitas et al [8] applied an 8-week BI to increase PADL and reported improvements in clinical asthma control, PADL, sleep quality, and anxiety symptoms in adults with moderate to severe asthma. Another effective strategy to improve the clinical control of asthma is the AT [13,14]. Furthermore, AT programs have been used as an intervention to reduce the exacerbation of asthma episodes and improve quality of life, making AT a treatment option [9].

**Objectives**

As previously mentioned, evidence suggests that AT or BI can improve asthma symptoms and reduce comorbidities. However, the comparison between these interventions in improving asthma control remains poorly understood. We hypothesize that these interventions will promote similar benefits in asthma control and quality of life in people with asthma. The main objective of this study is to compare the effects of AT versus BI in increasing physical activity for clinical asthma control. The secondary outcomes will include quality of life, physical activity level, sleep quality, asthma exacerbations, behavioral stages of physical activity, body composition, anthropometric variables, anxiety and depression symptoms, and cardiopulmonary exercise testing (CPET).

## Methods

### Study Design

**Figure 1:**
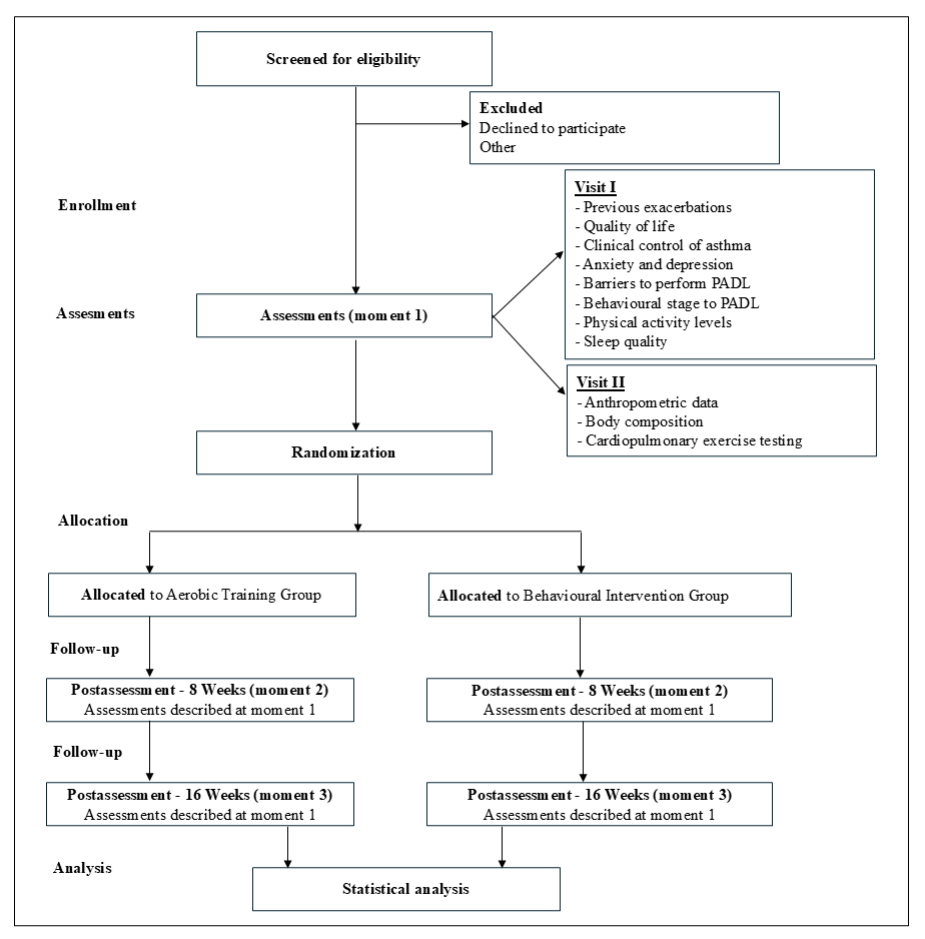
Study flow diagram of participant recruitment, randomization, interventions, and follow-up assessments. PADL: physical activity in daily life.

This study is a randomized trial with 2 parallel arms and a blinded evaluation. The study will be conducted at a university hospital that offers specialized treatment for moderate to severe asthma. The study protocol was developed in accordance with the SPIRIT (Standard Protocol Items: Recommendations for Interventional Trials) checklist guidelines [15], and the trial is registered at ClinicalTrials.gov (NCT05364632). The study flow diagram is presented in Figure 1.

After a regular medical visit, adult outpatients with asthma will be invited to participate in the study, and asthma pharmacotherapy will be maintained during the intervention. Initially, eligible patients will be randomized by a researcher not involved in the study using a computer-generated sequence into 2 groups: the AT group or the BI group. The total duration of each intervention for both groups will be 12 hours (720 minutes). Both groups will be evaluated before and after the interventions and again 16 weeks later. The evaluations will be carried out in a blinded fashion by an evaluator who will not be directly involved in the interventions.

### Participants

The study will include adults of both sexes aged 18 to 65 years with moderate to severe asthma [16] who are physically inactive (less than 150 minutes of moderate to vigorous physical activity per week) [17], and patients with uncontrolled asthma (Asthma Control Questionnaire [ACQ]≥1.5) [18]. In addition, the following inclusion criteria will be considered: diagnosis of asthma based on Global Initiative for Asthma recommendations [1], outpatient medical treatment for at least 6 months, and a stable clinical status for at least 30 days (no hospitalizations, visits to emergency services, or medication changes) [19].

### Ethical Considerations

Patients will be recruited from the pulmonology outpatient clinic of the Hospital das Clínicas de São Paulo, São Paulo, Brazil. This study will be conducted in accordance with the Declaration of Helsinki. The hospital research ethics committee approved the study (51929221.7.0000.0068). All participants will be informed about the possibility of withdrawing at any time without incurring any loss of treatment at the institution. Participants will receive an identification code upon inclusion in the study. The data will be deidentified by excluding the main identifying elements such as name, address, date of birth, phone number, email, medical record number, institutional identification number, and social security number. In addition, patients will be informed about the voluntary nature of their participation. Financial assistance for transportation will be available. Informed consent will be obtained by researchers from all study participants.

### Randomization

The randomization sequence will be computer-generated via an online platform (Sealed Envelope [20]) and implemented by an independent researcher who will be unaware of the sequence. The researcher will not be involved in recruitment, assessment, or treatment. As described previously, the randomization sequence will be concealed using opaque sequentially numbered envelopes [21]. Each envelope will correspond to one of the 2 study groups, and participants will be sequentially assigned to the envelopes after the initial assessments.

### Analysis of the Population

The exclusion criteria will include participation in another study, current smokers or ex-smokers (who stopped smoking less than 1 year ago or had a smoking history of more than 15 pack-years), other associated lung or heart diseases, a diagnosis of cancer in the last 5 years, difficulty understanding any questionnaires, pregnancy, or psychiatric problems that prevent an understanding of the questionnaires. If a patient misses any session, the session will be rescheduled. Attending at least 75% of sessions will be considered the cutoff point to establish patient compliance for both groups [22-24]. An intention-to-treat analysis will be performed using the patient’s most recent assessment in case of withdrawal from the study or absence of data [25].

### Interventions

#### Asthma Education Program for Both Groups

All people in both groups will participate in a brief asthma education program of 90 minutes. The educational program will be conducted in a class through group discussions and video presentations. Educational topics will include asthma pathophysiology, medication and peak flowmeter use, self-monitoring of symptoms, environmental hygiene [1,26], and physical activity recommendations and benefits [17].

#### AT Group

Patients will undergo aerobic physical training on a treadmill twice weekly for 16 sessions, totaling 8 weeks of intervention. Each AT session will last 45 minutes, divided into 5 minutes of warm-up, 35 minutes of aerobic exercise, and 5 minutes of cool-down (2 sessions per week, 45 minutes each). The intensity will be based on the heart rate (HR) corresponding to one third of the difference between the anaerobic threshold and the respiratory compensation point (RCP), and is determined by CPET [27]. After 2 weeks of adaptation, the exercise intensity will be increased to two-thirds of the difference between anaerobic threshold and RCP, obtained via the CPET. If a patient proves capable of sustaining the new training intensity uninterruptedly for 2 consecutive sessions, without presenting respiratory symptoms, the exercise intensity will be increased by 5% of the HR up to a maximum of 85% of the participant’s maximum HR [2,3]. Before and after each exercise session, patients will be asked to measure the peak expiratory flow, and if the value is less than 70% of the predicted value or if patients present important symptoms of asthma before or during training, they will be instructed to use 400 μg of salbutamol via aerosol prescribed by their physician [28].

#### BI Group

The BI will be based on social cognitive theory and the stages of behavior change to increase the practice of physical activity in people with asthma [12,29,30]. Strategies such as motivational interviews, feedback, and guidelines to overcome barriers will be used to make it easier for participants to reach their goals. Individuals will receive a commercially available smartwatch activity monitor (Mi Band 5; Xiaomi) with an alarm that vibrates when the recommended daily number of steps is reached and if the individual remains sedentary for 60 minutes [12]. The main objective of the BI for people with asthma will be to increase PADL at any intensity and reduce sedentary time. The program will be carried out in individual sessions once a week, lasting 90 minutes each, for 8 sessions. At the beginning of the protocol, a motivational interview will identify each participant’s behavioral stage for the practice of physical activity through an appropriate questionnaire [31]. In addition, the participants will be asked to complete a daily physical activity diary and sign a contract with their health care professional [12].

Data from each week will be reviewed at every session of the behavioral intervention. Each individual will receive advice based on the smartwatch data. Each session will address different topics related to the practice of physical activity and sedentary behavior. Individuals will be motivated and encouraged to make behavioral changes. In the last session, a final motivational interview will be carried out to identify the change in the patient’s behavioral stage, taking stock of the goals reached or not, the benefits acquired with the changes made, the strategies to overcome the most significant barriers, and the establishment of new long-term goals to stay physically active. The schedule and content of each session are detailed in Table 1, which is reproduced from Freitas et al [32]. The Freitas et al [32] protocol will be modified only by changing the activity smartwatch monitor (Mi Band 5; Xiaomi) during the intervention instead of the Fitbit device [32].

**Table 1:** Description of the behavioral change intervention sessions.

Number of weeks	Topics covered
Week 1: lifestyle choices	Motivational interview to establish an educational diagnosis. Identify the behavior stage regarding physical activity. Raise awareness of physical activity benefits. Provide the Mi Band 5 and ask them to wear it for at least 3 d each week.
Week 2: why become physically active?	Raise awareness of the physical activity international recommendations. Deepen the knowledge about the physical activity benefits for patients with asthma. Review Fitbit Flex 2 data of the past week and set one smart weekly goal (number of steps). Establish the action planning (goal) and sign a contract. Evaluate the confidence of patients in achieving the goal (self-efficacy). Explain about the use of the workbook, diary, and vibration alert.
Week 3: sedentary behavior	Raise awareness of the risks of prolonged uninterrupted periods of sitting. Ask them to start monitoring their sitting time (diary in a workbook). Discuss strategies to stand up/break up the sedentary time, according to the Fitbit Flex 2 vibration function. Review achievement (using the diary and Fitbit Flex 2 data) of the current goal. Discuss progress of the current goal. Set one smart weekly goal (number of steps).
Week 4: dealing with barriers	Dealing with barriers (as part of action and coping planning). Brainstorm the main barriers and possible solutions or modifications. Discuss preferred activities. Invite participants to come up with ideas for walking (progression in duration or intensity). Congratulate patients on any success (positive reinforcement) and ask them to reflect on any difficulties. Review achievement (using the diary and Fitbit Flex 2 data) of the current goal. Progress the current smart goal (number of steps and sedentary behavior).
Week 5: self-control	Facilitate self-control (how to self-monitor the negative and positive behaviors regarding PA^a^). Identify the benefits acquired with the lifestyle change and reinforce the commitment to change. Invite participants to come up with ideas to break up the sedentary time. Review achievement (using the diary and Fitbit Flex 2 data) of the current goal. Progress current goals as able or required.
Week 6: setting an additional goal	Review the initial goal and discuss the progress of this goal (challenges). Evaluate the confidence about achieving the new goal (self-efficacy). Reinforce the health benefits of increased participation in PA and of breaking up sedentary time. Congratulate on any success and reflect on any difficulties. Review achievement (using the diary and Fitbit Flex 2 data) of the current goal. Set a new smart goal as able or required.
Week 7: being rewarded	Identify the behavior stage regarding physical activity. Discuss the change (or not) that was achieved, as well as the benefits acquired with the new lifestyle. Discuss positive reinforcement. Review achievement (using the diary and Fitbit Flex 2 data) of the current goal. Set a final goal (number of steps).
Week 8: goal balance	Final motivational interview (goal setting, benefits acquired, and strategies to overcome barriers). Reinforce the importance of following through with these changes. Establish a long-term goal to stay physically active.

^a^PA: physical activity.

### Participant Timeline

The schedule of enrollment, allocations, interventions, and assessments is outlined in Table 2. The recruitment of the study participants began in August 2022.

**Table 2:** Schedule of enrollment, interventions, and assessments.

Study period			Enrollment (t1)		Allocation (0)		Baseline		After allocation (8 wk)		Follow-up (16 wk)
**Enrollment**											
	Eligibility screen	✓									
	Informed consent	✓									
	Allocation										
**Interventions**											
	Aerobic training			✓		✓					
	Behavior intervention			✓		✓					
**Assessments**											
	Lung function					✓					
	PADL^a^ and sedentary behavior	✓						✓		✓	
	Asthma clinical control	✓						✓		✓	
	Asthma exacerbation					✓		✓		✓	
	Quality of life					✓		✓		✓	
	Anxiety and depression symptoms					✓		✓		✓	
	Barriers to performing PADL					✓		✓		✓	
	Behavioral stage for PADL					✓		✓		✓	
	Anthropometric data					✓		✓		✓	
	Body composition					✓		✓		✓	
	Sleep quality					✓		✓		✓	
	CPET^b^					✓		✓		✓	

^a^PADL: physical activity in daily life.

^b^CPET: cardiopulmonary exercise testing.

### Outcome Measures

The primary outcome will be to compare the effects of AT and BI to increase the PADL on clinical asthma control. The secondary outcomes will include quality of life, sleep quality, asthma exacerbations, anxiety and depression levels, barriers that hinder the practice of PADL, body composition, and anthropometric data.

#### Primary Outcome: Asthma Clinical Control

Clinical asthma control will be assessed using the ACQ, a reliable and validated tool [18,33] that comprises 7 questions [18]. Of those, 5 questions are related to asthma symptoms (waking up at night, activity limitations, shortness of breath, and wheezing), one on rescue medication (short-acting β_2_-agonist use), and one on lung function (FEV_1_ prebronchodilator, expressed as a percentage of predicted value). The questions are indicated on a 7-point scale ranging from 0 (no limitation) to 6 (maximum limitation), and the total score is the average of the 7 items, ranging from 0 (fully controlled) to 6 (severely uncontrolled). It is considered that values ≥1.5 indicate poorly controlled asthma, 0.75 to 1.5 indicate partially controlled asthma, and ≤0.75 indicate fully controlled asthma [18]. A change of at least 0.5 points in the ACQ score is considered clinically significant [34].

#### Secondary Outcomes

#### Physical Activity in Daily Life

PADL will be objectively assessed using an activity monitor (ActiGraph GT9X, ActiGraph), a device that monitors frequency, intensity, and duration [35] in real time and is a sensitive and reliable method [36,37]. This triaxial monitor provides measurements for the amount and intensity of PADL and posture monitoring [35,38] through variations in acceleration. The “counts” obtained during a given period are linearly related to the intensity of the patient’s physical activity in that period [37]. Patients will be instructed to use the accelerometer for 7 consecutive days on the hip secured with an elastic belt. All accelerometers will be initialized to collect data in 60-second “epochs” in the 3 axes via ActiLife software (version 6.9.5; ActiGraph). The data will be analyzed using the same software, including the number of daily steps; the time spent engaging in sedentary behavior; and light, moderate, and vigorous activities. Moderate to vigorous physical activity will also be assessed.

#### Asthma-Related Quality of Life

Health-related quality of life will be evaluated using the Asthma Quality of Life Questionnaire [39,40]. This questionnaire consists of 32 questions related to the previous 2 weeks and is divided into 4 domains: activity limitation (11 items), symptoms (12 items), emotional function (5 items), and environmental stimulus (4 items). The Asthma Quality of Life Questionnaire score ranges from 0 to 7; a higher score will be associated with better quality of life. A difference of 0.5 points is considered clinically significant [41].

#### Sleep Quality

Sleep quality will be assessed via a monitor (ActiGraph GT9X, ActiGraph) worn on the participant’s nondominant wrist. The participants will be instructed to wear the device for 7 consecutive nights. The results for sleep latency (the amount of time required to fall asleep) and sleep efficiency (the number of sleep minutes divided by the total number of minutes the participant was in bed) will be analyzed [8]. The device is valid and reliable for detecting sleep/wake diurnal patterns [42]. In addition, participants will receive a diary for routine analyses covering these 7 nights (time participants went to sleep, time they woke up, and went to sleep on a scale of 0-10).

#### Asthma Exacerbation

Asthma exacerbation is defined as the patients’ need for urgent medical action and a change in pharmacological treatment [43]. During the study, at least one of the following criteria will be used to determine an exacerbation: the use of ≥4 puffs of rescue medication per 24 hours during a 48-hour period; the need for the administration of systemic corticosteroids (pills or injections); or unscheduled medical appointments, visits to the emergency room, or hospitalization [26,32,43].

#### The Behavioral Stages for Physical Activity

The “readiness for change” assessment will be graded through a questionnaire to assess the following behavioral stages for physical activity: *precontemplation*, *contemplation*, *preparation*, *action*, and *maintenance* [31,44]. Information will be revealed as frequency.

#### Barriers to Performing Physical Activity

Barriers to the practice of PADL will be assessed via a questionnaire that contains the most common barriers in adults [45,46]. The answer options for each question will include never, rarely, sometimes, almost always, and always, with the score for each item ranging from 0 to 4. Higher scores indicate greater barriers. At the end of the questionnaire, a question regarding the limitations of PADL resulting from the disease will be added, which will receive the same score as the previous questions.

#### Body Composition and Anthropometric Indexes

Octopolar InBody 720 equipment (Biospace) will be used to measure body weight, fat mass, fat-free mass, visceral adiposity area, and skeletal muscle mass. The InBody 720 uses 8 electrodes to assess body composition according to total body water, protein, mineral, and fat mass. The contact points for the electrode connection will be cleaned with an electrolytic cloth, according to the manufacturer’s instructions. At the time of testing, the patient should not be menstruating and will also be instructed not to ingest water for 4 hours and caffeine or alcohol for 12 hours before the test, or to perform physical activity or take a sauna the day before the test, and not to urinate 30 minutes before the test. The data will be electronically imported into Excel via LookingBody 3.0 software (Biospace). Anthropometric data, including height, body weight, abdominal circumference, waist and hip circumference, and waist-to-hip ratio, will be measured according to standardized protocols [47,48]. BMI will be calculated by dividing body weight (in kg) by height (in m^2^) [49].

#### Anxiety and Depression

The Brazilian Portuguese version of the Hospital Anxiety and Depression Scale will be used to assess the symptoms of anxiety and depression [50]. The Hospital Anxiety and Depression Scale consists of 14 questions divided into 2 subscales: anxiety and depression (7 questions each). Each question ranges from 0 to 3 points, with a maximum score of 21 points for each subscale. In this study, a cutoff score (≥9) will be used to classify the presence or absence of anxiety or depression [51].

#### Cardiopulmonary Exercise Testing

CPET will be performed via an electrical cycle ergometer (Corival; Lode BV Medical Technology) equipped with an electronic system (CPX System; CareFusion) [52]. Peripheral oxygen saturation (SpO_2_) and electrocardiography will be continuously monitored during the tests. The following variables will be recorded: work rate, oxygen consumption (VO_2_), minute ventilation, carbon dioxide production (VCO_2_), respiratory exchange rate, and HR [9]. Blood pressure, leg discomfort, and dyspnea will also be monitored using the Borg scale [53]. The participants will perform the CPET, which is limited by symptoms, consisting of 2 minutes of rest, 2 minutes of warm-up (unloaded pedaling), and a period of work on a ramp (from 5 to 15 W), with increments every minute, according to the participants’ daily activity level [9]. AT will be identified as the VO_2_ at which the change in the slope of the VCO_2_-to-CO_2_ ratio occurs. The RCP will be determined by the increased ventilation/VCO_2_ values, accentuated tachypnea, and a progressive reduction in end‐tidal CO_2_ pressure [9]. A 10% correction will be made in the HR at anaerobic threshold and RCP [54,55] because the test will be carried out on a bicycle, and training on a treadmill.

The test interruption criteria will include diastolic blood pressure above 140 mm Hg, sustained drop in systolic blood pressure, systolic blood pressure above 240 mm Hg, motor incoordination, feelings of imbalance, mental confusion, and clinical manifestations of respiratory distress exacerbated by increased workload or associated with electrocardiographic changes in ischemia and severe manifestations of exercise-induced bronchospasm, such as an excessive increase in ventilatory effort, audible wheezing, and signs of respiratory distress [56]. Patients will be instructed to use 400 μg of salbutamol via aerosols if necessary (peak flow, 70% predicted) [6,19,57].

### Statistical Analysis Planning

The sample size calculation (n) was established based on the clinical control measured by the ACQ to detect a clinical improvement of 0.5±0.6 points [34]. The required sample size will be 24 patients per group for a total of 48 patients, and a 15% loss will be considered, resulting in a total of 56 patients. The sample power was considered 80% with a significance level of 5%. Before the interventions, clinical, anthropometric, physical fitness, PADL, and psychosocial data obtained at baseline will be compared using the 2-tailed *t* test or Mann-Whitney test (depending on data normality) for continuous variables. The chi-square test will be used to compare categorical variables. After the intervention, time and group interactions will be evaluated using a two-way repeated measures ANOVA. The significance level will be adjusted at *P*<.05 for all tests, and the SigmaStat (version 3.5; Systat Software) program will be used for statistical analysis. All the analyses will follow the intention-to-treat principles until 15% of the data are missing. For confidentiality, the participants will be given an anonymous study ID, and only the study investigators will have access to the final trial dataset.

## Results

The clinical trial registration was approved in May 2022. Recruitment and data collection for the trial are ongoing, and the results of this study are expected to be completed by the end of December 2026.

## Discussion

### Potential Relevance and Impact of the Study

This randomized clinical trial, with 2 parallel arms and blinded evaluation, compares the effects of AT and BI on clinical control in individuals with moderate to severe asthma. The effects of physical exercise on people with asthma have been widely discussed in recent years [14], and physical exercise has been proposed as a nondrug therapy for asthma management. AT promotes anti-inflammatory effects in patients with asthma and improves exercise-induced bronchoconstriction [12]. In addition, physical training improves physical conditioning and health-related quality of life, and reduces bronchial hyperresponsiveness, the use of long-term bronchodilators and corticosteroids, and asthma symptoms [5,7,14,27,58,59]. BI, aimed at increasing PADL in people with asthma, also promotes better disease control [8]. Furthermore, recent studies have shown that a higher number of steps per day is associated with better clinical control of moderate to severe asthma [60], and that the minimum clinically significant difference is 1413 steps per day [61]. The results of this study may be highly relevant to clinical practice, as they will enable a similar understanding of how both interventions can improve clinical control of asthma in the short and medium term, regardless of the approach used.

### Strengths and Limitations of the Study

This study protocol will be a randomized trial with a 4-month follow-up postintervention. It is essential to determine whether the possible gains will be maintained in the medium term following the intervention. Objective measures of physical activity and sleep quality (ActiGraph GT9X), physical fitness (CPET), and body composition (Octopolar InBody 720) are another strength of this study. One limitation is that assessments using questionnaires are considered subjective measures. However, all tools have been validated and are widely used in interventions for people with asthma. The fact that the study will be conducted in a single hospital center may represent another limitation.

### Conclusions

To the best of our knowledge, this will be the first study to compare the effects of AT versus BI on increasing physical activity for clinical asthma control. Therefore, the results obtained in the proposed protocol may provide essential information for health care professionals when recommending these approaches to people with asthma. Additionally, this study may help clarify whether the potential short-term gains following the intervention are maintained in the medium term in people with moderate to severe asthma.

## Appendix

Multimedia Appendix 1 Peer-review report by FAPESP (São Paulo Research Foundation, Brazil).

## Abbreviations

ACQ: Asthma Control Questionnaire
AT: aerobic training
BI: behavioral interventions
CPET: cardiopulmonary exercise testing
HR: heart rate
PADL: physical activity in daily life
RCP: respiratory compensation point
SPIRIT: Standard Protocol Items: Recommendations for Interventional Trials
SpO2: peripheral oxygen saturation
VCO2: carbon dioxide production
VO2: oxygen consumption

## Data Availability

The datasets generated or analyzed during this study will be available from the corresponding author upon reasonable request.
