# Supplementary material for: Effects of Aerobic Training Versus Behavioral Intervention to Increase Physical Activity for Disease Control in Patients With Asthma: Protocol for a Randomized Trial
Source: JMIR Res Protoc. 2025 Oct 30;14:e78603. doi: 10.2196/78603 (PMC12616191; doi:10.2196/78603)
Supplement: Multimedia Appendix 1 [file resprot_v14i1e78603_app1.pdf]

FAPESP

FUNDAÇÃO DE AMPARO À PESQUISA DO ESTADO DE SÃO PAULO

## *REQUEST FORM FOR RECONSIDERATION OF GRANTS AND ASSISTANCE*
## *( Exclusive use by the SAGe system )*

| |
|---|
| PROCESS: 2021/03745-3 |
| BENEFICIARY: David Halen Araújo Pinheiro |
| ADVISOR/SUPERVISOR (when applicable): Celso Ricardo Fernandes de Carvalho |
| REQUEST FOR RECONSIDERATION no. (1, 2, 3...): 1 |

**1- INDICATE WHICH DOCUMENTS WERE ADDED TO THE CURRENT PROPOSAL**

| | | |
|---|---|---|
| ☒ REFORMULATED RESEARCH PROJECT | ☐ CURRICULAR SUMMARY | ☐ ACADEMIC RECORD |
| ☐ OTHERS: (specify) | | |

**2- ANSWER BELOW, POINT BY POINT, EACH QUESTION RAISED IN THE ADVISORY OPINIONS, INDICATING THE CORRESPONDING CHANGE IN THE PROPOSAL.**
**IMPORTANT : If there are changes to the research project motivated by the advisory opinions, they must be incorporated and highlighted in a reformulated research project, to be submitted to FAPESP with this request for reconsideration.**

We would like to begin by thanking the Advisors for their valuable comments and suggestions for the project. All suggestions were accepted, included in the project and marked in yellow. We forwarded the request for reconsideration regarding the PhD scholarship proposal and answered the questions point by point.

**Responses to the Opinion of the 1st advisory:**
We appreciate the Advisor's careful review and suggestions. We are forwarding the request for reconsideration based on the Advisor's glowing comments, which rated the advisor's research project and track record as "Excellent" and the candidate's track record as "Good." Below, we respond point by point to the Advisor's comments.

**Comment 1:** The history academic of the candidate is good (average) 7). Among the scientific articles published with factor of impact he has two articles as second author but no declaresno article as first author.
**Applicant:** We would like to thank the Reviewer for his/her comment. We would like to point out that the applicant has a study as first author in an international journal [1], where the authorship was shared **( the authors contributed equally to the study, information that is included in the article)** . In addition, the applicant has 4 (four) studies as first author published in national journals [2-5]. The applicant has 15 (fifteen) scientific articles published, 3 (three) in journals with an impact factor between 2 (two) and 3 (three). In view of this, we believe that we have shown that the applicant's academic record has adequate academic potential to obtain a FAPESP doctoral scholarship.

**References:**
1. **Campos R*, Pinheiro DHA*** , Britto-Junior J, Castro HA, Mendes GD, Moraes MO, Moraes MEA, Lopes-Martins RAB, Antunes NJ, De Nucci G. Quantification of 6-nitrodopamine in Krebs- Henseleit's solutive by LC-MS/MS for the assessment of its basal release from *Chelonoidis carbonaria aortae in vitro* . ***Journal of Chromatography B*** , 2021; 1173(22):122668. ***Both authors contributed equally to this work.** Impact factor = 3.0. Available at: https://www.sciencedirect.com/science/article/abs/pii/S1570023221001483?via%3Dihub .

2. **Pinheiro DHA** , Silva KKV, Araujo DMC, Sousa KS, Barros EML, Araujo KS, Carvalho AFM. Effects of laser alone and associated with swimming on lung inflammation in *rattus norvegicus* . ***Brazilian Journal of Surgery and Clinical Research*** , 2020;32(1):07.
3. **Pinheiro DHA** , Silva KKV, Silva CC. Effects of low-level laser in the treatment of cervicalgia: case studies. Varia **Scientia Health Sciences Journal** , 2018;4(1):86.
4. **Pinheiro DHA** , Silva MBM, Pinheiro DNA, Barros Filho EN, Lima ABM. Eating habits and health profile in children and adolescents in Teresina, PI. Varia **Scientia Health Sciences Journal** , 2018;3(2):106.
5. **Pinheiro DHA** , Garcez BBD, Lima ABM. Eating habits and lifestyle of students and teachers at a higher education institution in Teresina, PI. **FACID Journal: Science & Life** , 2016;12(1):66.

**Comment 2:** Presents historical academic with participation in projects of search outside this line of research, however has training scientific for driving of the theme of project PhD in this line of research. During your academic period developed initiation scientific, participated of extension activities, monitoring and awards. In summary the candidate declares authorship in 15 scientific articles having 03 articles with factor of impact between 2.0 to 3.0, being second and fourth author. The candidate presented one declaration of registration in level of master's degree to the Program of Postgraduate degree in Biomedical Engineering until December 2021.
**Applicant:** We appreciate the positive comments from the Advisor, which reinforce the applicant's scientific and academic background. We reiterate that the applicant is the first author of 5 (five) articles, one of which is in an international journal (impact factor 3.0) [1], in addition to co-authorship in another 2 (two) articles in international journals with an impact factor between 2 (two) and 3 (three) [2.3]. We hope that this information reinforces the academic potential of the applicant, who has just obtained his Master's degree (September 2021). We emphasize that the applicant's academic potential began in Scientific Initiation in the city of Teresina (Piauí), when he received an award and was awarded a scholarship to pursue his Master's degree in the State of São Paulo. However, due to issues that went beyond the applicant's wishes, the Master's degree, which would have been in the clinical area, ended up being pursued in the experimental area. However, the applicant, a physiotherapist by profession, has always wanted to pursue his postgraduate studies in the clinical area. For this reason, he is opting to change his PhD, despite all the opportunities he has been offered to remain in the experimental area. We also emphasize that the candidate has been participating in an internship in activities at the Hospital das Clínicas of FMUSP , including screenings and outpatient physiotherapy care for the population to be studied, since September/2021, when he completed his Master's degree .

**References:**
1. Campos R*, **Pinheiro DHA*** , Britto-Junior J, Castro HA, Mendes GD, Moraes MO, Moraes MEA, Lopes-Martins RAB, Antunes NJ, De Nucci G. Quantification of 6-nitrodopamine in Krebs- Henseleit's solutive by LC-MS/MS for the assessment of its basal release from *Chelonoidis carbonaria aortae in vitro* . ***Journal of Chromatography B*** , 2021; 1173(22):122668. ***Both authors contributed equally to this work.** Impact factor = 3.0. Available at: https://www.sciencedirect.com/science/article/abs/pii/S1570023221001483?via%3Dihub .
2. Britto-Júnior J, Jacintho FF, Campos R, **Pinheiro DHA** , Murari GMF, De Souza VB, Schenka AA, Mónica FZ, Moreno RA, Antunes E, De Nucci G. The basal release of endothelium-derived catecholamines regulates the contractions of *Chelonoidis carbonaria* aorta caused by electrical field stimulation . ***Biology Open*** , 2021; 1(10):057042. Impact factor = 2.0.
3. Britto-Junior J, **Pinheiro DHA** , Justo AFO, Murari GMF; Campos R, Mariano FV, De Souza VB, Schenka AA, Monica FZ, Antunes E, De Nucci G. ***Pharmacology Research & Perspectives*** , 2020; 4(8): e00612. Impact factor = 2.0.

**Dear Reviewer:** If you believe that your questions have been answered adequately, we kindly request that you improve the evaluation of the items evaluated. We are requesting this because we understand that the project and the researchers involved were well evaluated and that FAPESP's analysis is comparative in nature.

**Responses to the Opinion of the 2nd advisory board:**
We appreciate the Advisor’s careful review and suggestions. We forward the request for reconsideration based on the Advisor’s rating of the candidate’s academic record as “Good” and the advisor’s rating as “Excellent.” The research project was rated as “Very good, with some easily correctable deficiencies.” Below, we respond point by point to the Advisor’s comments and suggestions.

**Comment 1:** This is a two-arm randomized clinical trial with blinded evaluation whose main objective is to compare the effects of aerobic training and a behavioral intervention on several health outcomes such as physical activity level, quality of life, sleep quality, psychological disorders, exacerbations and barriers that hinder the practice of PADL in patients with asthma. The framework raised in the proposal indicates that both proposed interventions are beneficial to the study population but that they have not yet been tested in isolation regarding the outcomes presented. Overall, there is a good foundation, highlighting that part of the framework is repetitive although coherent and a deeper immersion and analysis of the cause-effect mechanisms could have been better explored to reinforce the proposal. Furthermore, if the results of the hypothesis are confirmed, that is, that they will have similar results, how should these results be applied in practice? What would be the potential contribution in this regard? Although implicit, it needs to be explored more clearly. I consider that the intended contribution is relevant and important.
**Applicant:** We are grateful for the comments and suggestions from the Assessor. We would like to highlight that a more in-depth approach to the cause-effect relationship of the interventions was explored and highlighted in yellow in the section: **"1.3 Effects of non-drug treatments"** . The practical application of the results was also included in the study hypothesis highlighted in yellow in the section **"1.4 Hypothesis"** , page 6.

**Comment 2:** Comments on the scientific basis were provided together with the previous item. In general, the methods used are consistent with what was intended and some points raised will be highlighted below. A consideration about the ACQ, in the order of the proposed methods is inconsistent since it is an inclusion criterion, it is also worth noting that several factors such as existing comorbidities will only be questioned according to what was indicated, which should be considered at the end of the study since in Brazil, the investigation of global health as well as of a preventive nature is not a reality for many patients who may be unaware of other health problems. The restriction on practitioners of aquatic physical activity and cycling is not very clear (are they not sedentary?) What is the rationale? Was sleep quality mentioned in an item as measured by the accelerometer? The assessment of body composition in a more detailed manner is not so evident in the basis and objectives. Is the application of the HAD related to the objective of psychological disorders? There is no established relationship in the proposal. In the cardiopulmonary stress test item, there is no mention of a severe EIB criterion for interrupting exercise, nor of whether or not the use of salbutamol is permitted during the test, nor is there any guidance for the days of exercise.
**Applicant:** We appreciate the critical comment from the reviewer. We apologize for not detailing some variables that are commonly used in our group and we ended up summarizing them due to the page limit established by FAPESP. Changes were made to the project to highlight the issues related to the ACQ, highlighted in yellow in section : **"3.3 Study design"** , page 8. We understand the concern about comorbidities, however, we emphasize that the Hospital das Clínicas of FMUSP is a tertiary care service that has specialized treatment for patients with severe asthma and all comorbidities are regularly investigated. This is because patients are followed for several years, which facilitates the investigation of comorbidities. A recent study by our group included 250 patients with severe asthma and demonstrated more than 15 (fifteen) comorbidities in our patients [1]. Regarding the comment "restriction of practitioners of aquatic physical activity and cycling", the expression was replaced by "physically active people or practitioners of regular physical exercise" **"3.2 Casuistry "** , page 8 and in **"3.3 Study design"** , page 10 ( Figure 1). Experimental design of the study). We also inform that sleep quality will be assessed using the ActGraph GT9X (ActiSleep monitor) as previously described by studies by our group and others [2-4]. Body composition was also better reported in section **"2.2 Secondary Objectives"** (page 7) as well as the application of HADS " **1.3 Effects of non-pharmacological treatments"** (page 6) and **"3.5.7 Symptoms of Anxiety and Depression "** (page 13). Finally, we inform that the interruption criteria and guidelines for performing the cardiopulmonary exercise test were described as requested by the assessor in section **" 3.5.9 Assessment of the physical capacity test "** (page 14) including guidelines for exercise days, medication use and quantification of peak expiratory flow: in section: **3.6 Interventions,** page: 15, highlighted in yellow.

**References** :
1. Freitas PD, Xavier RF, McDonald VM, Gibson PG, Cordova -Rivera L, Furlanetto KC, de Oliveira JM, Carvalho-Pinto RM, Cukier A, Stelmach R, Carvalho CRF. Identification of asthma phenotypes based on extrapulmonary treatable traits. **Eur Respir J.** 2021;57(1):2000240
2 . Lee, J.-M., Byun, W., Keill , A., Dinkel, D., & Seo , Y. Comparison of Wearable Trackers' Ability to Estimate Sleep. **Int J Environ Res Public Health** . 2018; 15(6):1265.
3. Sadeh A. The role and validity of actigraphy in sleep medicine: an update. **Sleep Med** Rev. 2011; 15(4):259-267.

4. Freitas PD, Silva AG, Ferreira PG, Silva A, Salge JM, Carvalho-Pinto RM, Cukier A, Brito CM, Mancini MC, Carvalho CRF. Exercise Improves Physical Activity and Comorbidities in Obese Adults with Asthma. **Med Sci Sports Exercise** . 2018 ;50:1367 -1376

**Comment 2:** The student is scheduled to defend his master's degree in June 2021 and start his doctoral activities, signed by his advisor, in August 2021. The activities of this proposal will be developed over a period of 7 semesters (from 2nd semester 2021 to 2nd semester 2024), with 5 semesters dedicated to data collection. The proposal involves several assessments and intervention, understanding that there will be a need for support, but there is no mention of this. The required sample size will be 24 patients per group ( total = 48; loss of 15% = 56 patients). There is an intention to start in-person data collection in 1st semester 22, however, there is no certainty about the maintenance and adherence to these activities if the pandemic still exists. There is no mention of the intention of internationalization and mobility, which will also depend on health conditions and rules for opening borders. In general, if the pandemic conditions allow, there is feasibility of execution.
**Applicant:** We understand and appreciate the Advisor 's concern regarding the current health aspect, as well as the support given to the applicant in carrying out the tests and assessments. Regarding support, we inform that our research group has financial resources from the FAPESP thematic project (2018/017788-3) which has technical support grants (TT-3) that will be used to assist the progress of the project. We also emphasize that the Hospital das Clínicas has resumed all its activities as normal due to the drastic reduction in COVID-19 infections in the last 3 months and that the level of infection and death is also at the lowest levels in the last 24 months. In addition, all infection prevention protocols will be duly adopted and the activities will be carried out with the hygiene care required by the Infection Control Committee (CCIH) of the Hospital das Clínicas. Regarding internationalization, both the applicant and the advisor are fully interested, however, this will depend on issues that, at this time, we are not yet able to confirm. We are communicating with researchers at Newcastle University (Australia), which has a center of excellence in severe asthma, and with whom the supervisor has established collaboration on a project with patients with severe asthma (https://treatabletraits.org.au/staff/celso-carvalho-fernandes-de-carvalho/)

**Comment 4:** Candidate with insufficient experience in the research area in which he/she is involved but with training in Physiotherapy and the possibility of learning the needs according to the schedule.
**Applicant** : We understand the advisor's concern. We would like to point out that the applicant is a Physiotherapist who is currently studying in Teresina (Piauí), where he has done his scientific initiation with distinction, won an award and received a scholarship to complete his Master's degree in the state of São Paulo. He was interested in completing his Master's degree in the clinical area, also because of his professional training. However, for reasons beyond the applicant's control, he ended up having to complete his Master's degree in the experimental area. However, at this time, the applicant wants to return to clinical activities related to his undergraduate degree in Physiotherapy. We would like to reiterate that the candidate's potential stands out to such an extent that, even though he is not in the experimental area, he has published several articles. In addition, the applicant has received several invitations to remain in the experimental area and is giving up several benefits just to return to the clinical area. Additionally, the applicant has been performing an internship to monitor the group's activities, including screenings and outpatient physiotherapy care for asthma patients since September/2021 at our outpatient clinic at Hospital das Clínicas of FMUSP. Therefore, the applicant already has experience with the patients and protocols to be applied in this proposal. We confirm that the applicant is already monitoring the care of other studies in the same population.

**Dear Reviewer:** If you believe that we have responded adequately to your comments, we kindly request that you improve the evaluation of the items evaluated. Our request is made because we understand that the project and the researchers involved were well evaluated and FAPESP's analysis is comparative in nature .

**3- ADD COMMENTS THAT YOU CONSIDER IMPORTANT FOR THE ANALYSIS OF THE RECONSIDERATION**

**(It is worth remembering that, if, for any reason, it is deemed necessary and appropriate to contest the quality of the advisory opinion or the adequacy of the advisory itself, this should be done in a separate letter, addressed to the Scientific Director, justifying the request that a new advisor be heard)**

**4- PLACE, DATE**

Sao Paulo, SP, November 26, 2021.

| | |
|---|---|
| **Provision of Accounts (Dates)** | 10/02/2023<br>10/02/2024<br>10/02/2025<br>10/09/2025 |
| **Allows extension from the Bag?** | Yes |

## Observations / Transcriptions / Phrases

**Observations to the Responsible**

We communicate what your request of bag, constant of process above referred to, he was analyzed and **approved** by FAPESP.

Put please, wait the e-mail with to the instructions to confirmation of interest for the concession.

To knowledge of content of dispatch, put please, access the System SAGe ( www.fapesp.br/sage), selecting the menu item My Processes>>Process Number and, under More Information, the option Dispatch.

With exception of bag of Post PhD, the transcription of opinion this available exclusively to the advisor, who is responsible for choosing the excerpts to be shared with the candidate.

To any consultation or communication on this correspondence, put please, use exclusively you services from "Talk to FAPESP" at www.fapesp.br/converse.

Yours sincerely,

Louis Eugene THE. M. Mello
Scientific Director

PENDING

- Proof of registration in the doctorate

**Phrases to the Responsible**

*No there is phrases associated.*

**Transcription of Opinion to the Responsible for the Coordination Opinion**

Proposal recommended.
THE analysis collegiate from the FAPESP, having as basis the opinion of the consultancies, considered what the proposal reformulated, it is now well qualified in its main aspects and reaches the level of excellence necessary for recommendation.
------------------

OPINION ADVISORY 2:

**APPRECIATION GENERAL FROM THE PROPOSAL**
------------------
**THE Fapesp denomina "Proposal" the set of three parts the to be evaluated, compound put: 1. Project Research History; 2. Candidate's Academic History; and, 3. Advisor's Research History. Please complete this item after completing the rest of the form.**

------------------
**1. Put please, analysis the PROJECT OF SEARCH proposed, according to road map below:**
------------------
**1.a. Analysis the definition, the relevance, the originality of the objectives and the importance from the intended contribution to the area of knowledge in which the proposed project is inserted.**

I keep the assessment of what this proposal it presents relevance and importance from the contribution intended. In this reconsideration analysis, changes were made in response to the advisory surveys. For the research project item, the content of the introduction had previously been identified as being very repetitive and lacking in depth, especially regarding the physiological explanations and detailed effects of the proposed interventions. There was also a change in the hypothesis item. Although a paragraph was added, this item was not substantially changed.
------------------
**1.b. Analysis the basis scientific and you methods employees.**

Overall, I maintain the opinion that the methods employed are consistent. For reconsideration analysis, details methodological they were provided and others enlightened and introduces itself better alignment basis, objective and methods used.
------------------
**1.c. Analysis the adequacy of project the one program of PhD and feasibility of your execution within of**

**term expected.**

THE student and today teacher in Engineering Biomedical and there was modification of timeline to start of the activities in 2022 (1st semester) with completion scheduled for 2025. This reconsideration responds to an aspect of concern raised put this Consulting referent to the activities and the pandemic. Candidate informs what to the activities had been resumed in reason from the fall of the cases and protocols of contingency respected. In this At this time, the pandemic is once again showing a significant increase, which can be consulted at an appropriate time. The intention of internationalization is reported. From what is observed, there is project adequacy and feasibility of execution.

------------------

**1.d. Case if treat of one candidate what already you have started the doctorate, rate the progress of project research and the feasibility of its execution within the expected timeframe.**

There was no declaration of the beginning of the doctorate, but the defense of the master's dissertation is noted in the transcript. Again I inform what in terms temporal there is feasibility the closing predicted of the activities it will be in 2025

------------------

**1.e. THE Flat of Management of Data describes you types of data the to be produced for the project, to the ways of storing, preserving and sharing it.**

Assessment maintained: A data management plan was presented describing the data to be obtained, the form of storage, preservation and sharing what he meets to the requirement. You data will be made available in the scientific data repository of the University of São Paulo (https://uspdigital.usp.br/repositorio/.

------------------

**Conclusion on the analysis of Project of Search presented. (Filling mandatory)**

[ ] Excellent
[X] Very good
[ ] Very good, with some deficiencies easily curable [ ]
Good
[ ] Good with
deficiencies [ ] Regular
[ ] With serious deficiencies

------------------

**2. Please review the CANDIDATE'S SCHOOL AND ACADEMIC HISTORY. Doctoral Scholarships are intended for the students with good performance academic evaluated, mainly, for the historical undergraduate and graduate school. Granting to students with irregular academic records, showing a pattern of failures or approvals with minimum grades, is only possible in exceptional circumstances in view of other evidence of the candidate's academic potential, such as a successful Scientific Initiation/Master's project. Analyze according to the script below:**

------------------

**2.a. History School of candidate.**

The candidate completed the Physiotherapy course in 2018, without failing any subjects. The transcript does not indicate the grade for approval and average/performance coefficient. The candidate completed the activities in the master's degree/Univap (Program of Postgraduate studies in Engineering Biomedical) in 2021 and it presents the historical updated with the defense. There is no official document of the beginning of the doctoral course.

------------------

**2.b. History Academic of candidate (participation in projects of search, bags previous, scientific publications, awards).**

Assessment maintained without supply of new documents
THE document forwarded demonstrates what the candidate he was Scholarship holder of Program of Initiation Scientific and Technological by 3 times, monitor of disciplines and extension. There is mention of two awards without clarity on the study and authorship position. Collaborator of 15 scientific studies published in journals, 3 of which are international with the greatest impact (in two occupies the second position). Inform participation in others activities academics as events scientific, monitoring and lectures. There is no information on co-supervision of students. It should be noted that the research engagement theme to date is not directly related to the current request.

------------------

**2.c. Others items what compose the description of the activities academic, scientific and professionals developed by the candidate.**

------------------

**Conclusion on the analysis of History School and Academic of Candidate. (Filling mandatory)**

[ ] Excellent
[ ] Very good
[X] Good
[ ] Regular
[ ] With serious deficiencies

------------------

**3. Put please, analysis the HISTORY OF SEARCH DO ADVISOR, following the road map below:**

------------------

**3.a. Quality, regularity and importance of scientific and/or technological production, with emphasis on recent production. Important elements for this analysis are: list of publications in journals with selective editorial policy; books or book chapters; patents in which the inventor appears; other instruments of property intellectual; results of search effectively transferred and adopted by companies or the government; and other information that may be relevant.**

Assessment Maintained

The advisor declares in his list of publications in journals with selective editorial policy that he has a total of 124 publications, in addition to published chapters demonstrating regularity and importance of scientific production. From the list of articles listed in your summary it presents all in years recent as author reference. Average of quote Web of Science/Publons: 17.1 citations per article
Leadership: 93% of the publications they are as first or last author. Amount of quotes received: Web of Science/Publons=2.186 (H-Factor=26); Scopus=2.403 (H-Factor=26); Researchgate=3.150 (H-Factor=31); Googlescholar=6.132 (Factor H=40);O advisor got the title of doctor node year of 1996 and Free Teaching in Cardiovascular Physiotherapy -UNESP in 2011.

------------------

**3.b. Experience and competence demonstrated in the leadership of projects of research related to the theme of the proposal under analysis.**

Assessment Maintained
The advisor declares several research funding projects and grants. Research Productivity Grant Holder: Agency: CNPq - Level 2; Project thematic FAPESP 2018/17788-3: Start Jan/2021. All you projects encompass the theme presented in this current proposal.

------------------

**3.c. Experience International in search after the PhD or to have demonstrated participation active in international research collaboration networks.**

Assessment maintained
THE advisor declares partnerships international, publications joint and Visits.

------------------

**3.d. Capacity demonstrated to form researchers, with emphasis to the activity recent student guidance.**

Assessment maintained
THE advisor declares 05 supervisions of postdoctoral; 33 dissertations of master's degree; 11 guidelines of PhD with excellent demonstrated ability

------------------

**3.e. Results obtained for the advisor with financing previous from the FAPESP.**

Assessment maintained
THE advisor declares in document specific, significant amount of products resulting attached the research projects and grants from this agency with high impact

------------------

**3.f. Availability to guidance considering the regime of work and the number total of students under his guidance.**

Assessment maintained
No there is clarity this information. There is mention of 02 guidelines of master's degree, 01 initiation scientific and 01 PhD with scholarship support. There is no information about other students without support.

------------------

**Number current of mentees.**

1 IC
2 MS
0 DD
1 DR

------------------

**Regime of work.**

Assessment maintained
Bond Current: FM -USP, Regime of of Dedication Integral the Teaching and the Search

------------------

**Comments**

------------------

**3.g. Others considerations on the production scientific, technological and academic of advisor, relevant to the Analysis of the feasibility of the proposal.**

------------------

**Conclusion on the Analysis of History of Search of Advisor. (Filling mandatory)**

[X] Excellent
[ ] Very Good
[ ] Good
[ ] Regular
[ ] With serious deficiencies

------------------

**4. DEFICIENCIES NOTED (If some item you are marked, to the reasons must be indicated in the corresponding box of the form)**

------------------

**4.a. On the Project of Search, according to indicated node item 1::**

[ ] Project with objectives bad defined, excessive or incongruent. [ ]
Project with excessively limited objectives.
[ ] Project little original.
